# Bioinformatics Resources for Plant Abiotic Stress Responses: State of the Art and Opportunities in the Fast Evolving -Omics Era

**DOI:** 10.3390/plants9050591

**Published:** 2020-05-06

**Authors:** Luca Ambrosino, Chiara Colantuono, Gianfranco Diretto, Alessia Fiore, Maria Luisa Chiusano

**Affiliations:** 1 Department of Agricultural Sciences, University of Naples Federico II, 80055 Portici (Na), Italy; luca.ambrosino@szn.it (L.A.); chiara.colantuono@szn.it (C.C.); 2 Department of Research Infrastructures for Marine Biological Resources (RIMAR), 80121 Naples, Italy; 3 Italian National Agency for New Technologies, Energy and Sustainable Economic Development (ENEA), 00123 Rome, Italy; gianfranco.diretto@enea.it (G.D.); alessia.fiore@enea.it (A.F.)

**Keywords:** genomics, transcriptomics, proteomics, metabolomics, data integration, stress

## Abstract

Abiotic stresses are among the principal limiting factors for productivity in agriculture. In the current era of continuous climate changes, the understanding of the molecular aspects involved in abiotic stress response in plants is a priority. The rise of -omics approaches provides key strategies to promote effective research in the field, facilitating the investigations from reference models to an increasing number of species, tolerant and sensitive genotypes. Integrated multilevel approaches, based on molecular investigations at genomics, transcriptomics, proteomics and metabolomics levels, are now feasible, expanding the opportunities to clarify key molecular aspects involved in responses to abiotic stresses. To this aim, bioinformatics has become fundamental for data production, mining and integration, and necessary for extracting valuable information and for comparative efforts, paving the way to the modeling of the involved processes. We provide here an overview of bioinformatics resources for research on plant abiotic stresses, describing collections from -omics efforts in the field, ranging from raw data to complete databases or platforms, highlighting opportunities and still open challenges in abiotic stress research based on -omics technologies.

## 1. Introduction

Plants display an amazing diversity and, owing to their sessile nature, they evolved a broad range of molecular mechanisms to respond to complex network of environmental signals, which activate multiple pathways, modulated by different responsive genes, in case conferring tolerance to the pressure determined by stressor factors [1,2,3]. Abiotic stresses, such as heat and cold, drought, salinity and flooding, [4,5,6,7], however, dramatically affect plant growth and crop yield [2,8,9,10,11,12,13,14,15,16], and these are among the reasons why abiotic stress management is one of the most important challenges in agriculture. In current climate change scenarios, exposure to abiotic stresses is more frequent and the consequent effects are so relevant also considering the exponential increase of the world food supply due to the rapid population growth [2,9,12,13,17,18,19,20,21], and the widespread attention to promote a sustainable productivity. This is why extensive studies have been focused on understanding the molecular basis of abiotic stress response and the research for improved, productive plants, adapted for stress tolerance [10,13,20,22]. These activities were strongly favored by the evolving -omics technologies, which provide key strategies to promote molecular investigations on plant organization and functionality, also under stress conditions [23,24,25,26], and novel approaches for omics assisted crop improvement [27,28]. Since their initial introduction, they permitted unexpected views on different levels of cell functionality, ranging from genome to transcriptome, to proteome and metabolome, and more recently covering also investigation on chromatin organization by epigenome approaches [29,30,31,32].

These approaches, covering different levels of biological functionalities, enabled deeper investigations at each level as well as integrated views [33,34,35] to study the complexity of the molecular response of plants, and to abiotic stresses as well. Moreover, the technological evolution and cheaper methodologies offered faster and more accessible approaches favoring researches considering an increasing number of crops [36,37]. The so-called “Next Generation Sequencing” (NGS) technologies, as one of the major examples, largely favored deeper insights on plant genome organization [38,39,40,41,42,43] and on functional responses to variability of environmental parameters, elucidating the first level of gene expression, i.e., the transcriptome analysis, by promoting the transition from expressed sequence tags (ESTs) and microarray based techniques [44,45], to more powerful approaches such as RNA-seq [46,47,48] and associated technologies [29,49,50].

Simultaneously, the development of proteomics procedures by 2D-Gels coupled to mass spectrometry (MS) [51] or, more recently, via high-throughput shotgun approaches [52], and of robust LC–MS (liquid chromatography-mass spectrometry) [53] and GC–MS (gas chromatography-mass spectrometry) [54] metabolomics technologies, able to unravel fluctuations of non-volatile and volatile metabolites, are paving the way to a better understanding of the effects of the biological processes under investigation [33]. In this context, the integration of results from different levels of molecular information favors holistic views to decipher key components that are playing roles in complex molecular processes involved in plant responses to unfavorable or changing environmental conditions [55,56,57].

Bioinformatics is necessary for data production in support of the different omics technologies, fundamental for data organization and for data mining. It favors the interpretation of the massive amount of information provided by high throughput technologies, permitting the filtering of valuable information for human driven interpretation and assisting single level approach and data integration for comprehensive views on systems functionality [34,35,58].

Moreover, bioinformatics also provides overwhelming amount of accessible resources to the scientific community, driving pioneering research based either on the exploitation of -omics technologies [33,59,60,61] or of the manifold resources that may support specific subsequent analyses, such as those based on sequence comparisons, gene family investigations and molecular modeling [62,63,64].

Bioinformatics resources implementation and maintenance were among the main drivers of the success of this research field, and of the evolution of the omics technologies, since the data exploitation revealed to be a very powerful approach to support the overall scientific community. One of the key points to this aim remains the making of data accessible, reliable and suitable to be compared, touching the new challenges in the field, which fall in the so called integrative bioinformatics [65]. However, data exploitation is today still relying on scientist consciousness about the opportunities and limits offered by the different data sources, about the sensitivity and specificity of the different technologies, and about the quality of the organized results. Additionally, inexpert users must be aware of basics from the field to profitable handle, analyze and compare data from the available resources, to obtain novel insights into the organization and functionality of the biological systems.

To this aim, in this review, after a brief introduction of the main technologies that are accompanying the production of massive molecular data, we provide an overview of major bioinformatics data resources available in support of plant research, and specifically on abiotic stress responses, ranging from raw collections, to complete databases or platforms. We describe their main features, their usefulness and some of the bottlenecks that highlight the need of coordination in the efforts as well as education in data and software handling, for a suitable exploitation, to support the detection of key structure/functional features to interpret molecular processes involved in plant responses to abiotic stress conditions [24,46,66,67,68,69]. We will also describe the open challenges to increase and improve the opportunities offered by bioinformatics for innovative and effective research in the field.

## 2. Genomics

Genome sequencing has dramatically evolved in the last years, moving from BAC-by-BAC (also known as clone-by-clone shotgun strategy) [70] to whole genome shotgun (WGS) approaches [71], favored by novel technologies and, in particular, by the introduction of the next generation sequencing (NGS). The possibility of sequencing billions of fragments of DNA sequences in parallel, exploiting cheap and fast high-throughput technologies in place of the well-established Sanger technique, incredibly changed the scenario, making genome sequencing feasible and accessible even to not experienced laboratories [72]. This favored the spreading of several genome-sequencing efforts [73] moving from the sequencing of a limited number or reference model species [74,75,76,77,78,79] to the release of a multitude of draft genome sequences from different species and genotypes [80,81,82,83,84,85,86], often associated to preliminary gene annotations [87,88] of a variety of plant species and genotypes. These efforts provided reference collections, expanding the number of available resources for the same species or for representatives of under investigated clades [36,37,89,90,91,92,93,94]. These efforts are revealing fundamental molecular information useful also in plant breeding practices. Consequently, web accessible reference resources also flourished to collect the data and offer their value added information, as derived from their processing, to all the interested community. Beyond the centralized support offered to this aim by reference centers like the EMBL-EBI sequence collection [95], the DNA Data Bank of Japan (DDBJ) [96] and the National Center for Biotechnology Information (NCBI) [97], which acts as part of the International Nucleotide Sequence Database Collaboration (INSDC) [98] and collects results from worldwide sequencing efforts, maintaining these huge amount of results in dedicated partitions [99,100], several plant specific resources are also today available. In Table 1, some of the main reference sites, together with their current content (updated on February 2019) in terms of available gene annotation version for reference plant species, are reported. Among them, Ensembl Plants [101] is an integrative European resource for mining, visualizing and analyzing genomics data, currently collecting information for 44 plant species. Two additional interesting platforms are PlantGDB [102], containing data and tools for plant genomics, and reporting genomes of 27 plant species, and Phytozome [103], the plant comparative genomics portal of the Department of Energy’s Joint Genome Institute, which includes information on 52 plant species. Other platforms with more specific aims in terms of included species and offered services are also available. As examples, Plaza [104] is a comparative genomics resource providing information useful to investigate gene and genome evolution in plants, TreeGenes curates genomic and phenomic information for 1964 species [105], Gramene is a curated data resource for comparative functional genomics in crops and model plant species [106]. A reference multi-integrated platform is The Arabidopsis Information Resource (TAIR) [107], a representative example of a plant species-specific database that is proposed as a multilevel platform, i.e., it contains data from different levels (i.e., transcriptomics, proteomics, etc.) and methodological approaches (e.g., microarray, RNAseq, etc.) exclusively dedicated to the model plant *Arabidopsis thaliana*. Other interesting examples of dedicated platforms are for Solanaceae, one of the more investigated plant family, due to their relevance as crops (tomato, potato, eggplant, and pepper), for basic research and for industrial interest (tobacco and petunia): for this reason, massive achievements have been accomplished to improve their knowledge at molecular level in these last years. In this context, The Sol Genomics Network (SGN) [108] is a clade-oriented database dedicated to Solanaceae and closely related genomes. A similar effort is from Spud database [109], which is considered a reference resource for potato genomic data, a member of the Solanaceae too. Other examples of dedicated resources are: MaizeGDB [110], the Legume Information System [111] and Soybase [112].

Due to the spreading of these activities, parallel efforts are in progress to organize all related plant genome resources, and differences in terms of data release versioning are evident even in reference platforms such as Ensembl Plants [116] and NCBI [99] (Table 1). This heterogeneity, which often may also refer to circulating genome assemblies and/or gene annotation versions, poses a relevant issue in terms of the quality of the different resources and of reliability as well as comparability of the results they may provide. Indeed, the lack of uniformity is a natural consequence of the fast evolving performances of sequencing technologies, which are the core of the incredible acceleration of sequence data production at affordable costs, but the fast production and release of novel genome assemblies affects the establishment of good quality references [88]. Moreover, the constant release of novel or updated sequenced genomes encounters the bottlenecks of the lower efficiency in performing the consequent analyses, such as gene annotation, resource updating and curation, often affecting the maintenance and the updating of accurate information even in reference platforms. Indeed, evolving bioinformatics need to accompany the fast development of novel technologies and to constantly adapt to larger data numbers of increasing complexity, to favor the sharing of reliable information both for single species analyses and for comparative efforts. As an example, assembly quality needs clear assessments, while annotation versions should be reconciled. Moreover, the poor quality of protein coding regions predictions, especially in preliminary gene annotations, should require suitable methods for gene based comparative analyses and to appropriately define orthologs or paralog relationships [117,118].

Although novel strategies are being introduced to face the big data challenge in bioinformatics [73,119,120,121,122,123], the information quality assessment and the spread of well-established references requires longer-term efforts and validations to favor their dissemination to the scientific communities and fruitful subsequent exploitations. This is essential since reliable and stable references are also fundamental in order to appropriately exploit the resources in associated efforts, such as those from transcriptomics, epigenomics, proteomics and metabolomics [58,88].

Further challenges are determined by possible limits in data functional annotation. Although well-known efforts are engaged to provide suitable description of gene functionalities, such as the one sustained by the gene ontology consortium (GO) [124], or exploiting further appropriate information to integrate the analysis [125,126,127] they need to evolve according to the knowledge acquired on a specific species, since the available descriptions are limited by the available observations [128].

Knowledge-dependent collections may generate a further source of heterogeneity due to the availability of different resources, although often related by similar content.

As an example, querying the NCBI Gene database, a collection of gene-specific information from multiple data sources [129], by the simple “abiotic stress” keyword results in a large set of genes, which contain the words “abiotic stress” in their functional annotation (Table 2). In the same table, we also report the result of the same query made in the NCBI Refseq database, which provides integrated and curated collections, with non-redundant and well-annotated sets of sequences [99]. Provided that non-specific keywords could bias the search and it would be more appropriate to use more stringent keywords, e.g., “salt stress” or “drought stress”, the different results show that the same reference site can deliver different pieces of information. In fact, the same reference site can deliver different pieces of information, and this holds even when using more specific keywords, like, as an example, “drought stress” (Table 2). Of course, there are technical explanations that justify the discrepancies between the two query results, which are mainly related to their specificities, which are also described and well-known to experts. The ambiguous information from distinct resources can confuse non-experts, compromising the subsequent analyses. Moreover, the heterogeneity in the results between different plant species, e.g., the number of *Arabidopsis thaliana* abiotic stress-related genes compared to other species (Table 2), proves that comparative efforts are still highly required to transfer the assessment of gene roles from reference models to other species.

Bioinformatics plays a crucial role also in supporting novel challenges for plant improvements. As a recent example, the genome editing through the CRISPR/Cas9 approach [130], which can be also exploited for applications in abiotic stresses [131], strongly relies on accessible genomics knowledge and appropriate development of bioinformatics tools. In particular, several software have been developed to predict and select CRISPR/Cas9 targets for genome editing, such as the web based tools E-CRISP [132], TIDE [133], CHOPCHOP [134] and CCTop [135].

## 3. Transcriptomics

Comparison of transcriptome data is highly exploited to understand gene functionality and expression mechanisms in different tissues in various conditions [136,137,138]. Due to the general importance of transcriptomic data to depict gene expression patterns and to infer on gene control and regulatory mechanisms of cell functionality, the processes involved in plant responses to abiotic stress are being largely investigated at the transcriptome level through microarray and RNA sequencing analyses, such as expressed sequence tags (ESTs) and RNA-seq [32,44,139,140,141].

Microarray technology has been one of the most used approaches to explore the transcriptional landscape of a biological sample [142]. With the development of the GeneChip ATH1 Genome Array for *A. thaliana*, probe sets for around 24,000 genes were designed [45], disseminating the use of microarray analyses in plant sciences. This reference array facilitated the collection of results from multiple tissues and conditions and, as a consequence, the organization of gene expression atlases for *Arabidopsis*, which were also associated to useful computational strategies in resources that favor gene coexpression analyses [143].

The ArrayExpress Archive [144] and the Gene Expression Omnibus (GEO) [145] are main public collections of functional genomics data currently available. Querying the two platforms by the “abiotic stress” keywords resulted in 436 and 157 array experiments in *GEO* and ArrayExpress databases, respectively (Table 3).

**Figure 1:**
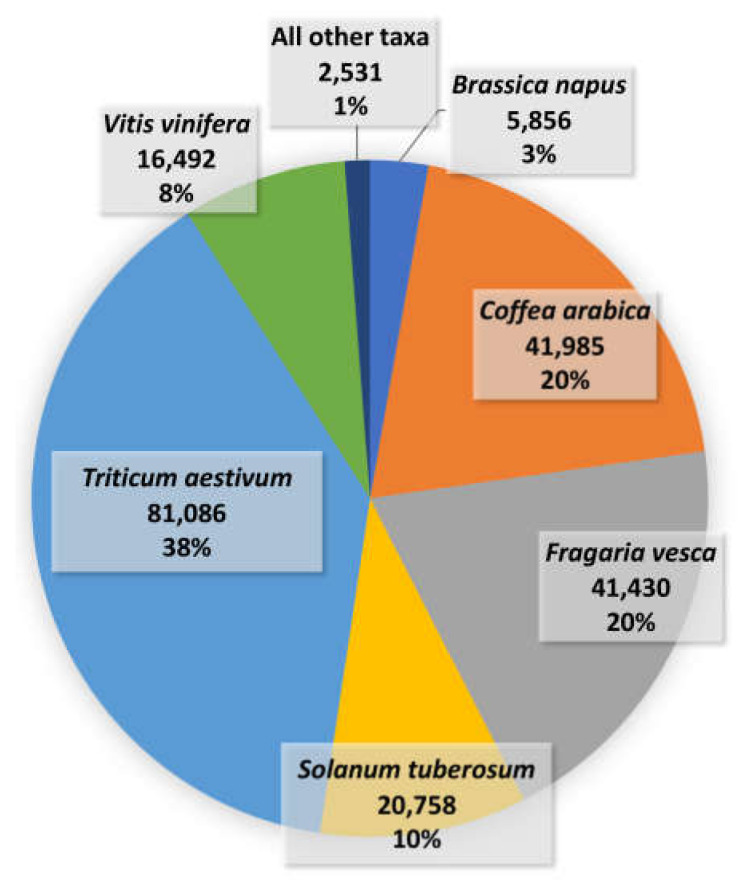
“Abiotic stress”-related expressed sequence tags per each plant species available in dbEST database.

Expressed sequence tags (ESTs) are sequences derived from randomly selected complementary DNA (cDNA) libraries [146]. dbEST at the NCBI [147] is the specialized division of GenBank, which contains all publically available EST collections from different organisms, currently delivering 221,400 ESTs referring to a total of 77 different libraries, when queried by the “abiotic stress” keywords in viridiplantae (Figure 1 and Table 4). EST sequencing has been recently widely replaced by the spread of cheaper and more powerful techniques based on NGS technologies, such as RNA-seq and small RNA-seq [48], to define and quantify the RNA abundances in a cell [148], or in a specific tissue and/or in specific conditions. Microarrays, the most widely used transcriptomics technology for many years, are being replaced by RNA-seq too, which is becoming the dominant technology today [47,48], due to the higher sensitivity and cost accessibility. Although RNA-seq analyses that rely on already sequenced and assembled genomes provide robust results [47,48,149,150,151,152,153,154,155], RNA-seq suitably supports transcriptome-based analyses also of non-model species [156,157,158,159,160,161,162,163,164,165,166,167,168]. The knowledge of the analyzed genome is not a prerequisite for the analyses, providing a consistent representation of the transcriptome content in comparison to the microarray technology, thanks to its powerful throughput.

The NCBI Sequence Read Archive (SRA) [100] is the reference resource established in order to gather public collections from worldwide NGS efforts, including data from Illumina Genome Analyzer [169], Applied Biosystems SOLiD System [170], Roche 454 GS System [171] and Helicos Heliscope [172]. Currently, SRA reports 330 accessions from 26 plant species when querying for the “abiotic stress” keywords in viridiplantae (Table 5).

Beyond general reference resources, numerous efforts aimed to the set-up of species specific or clade specific databases, over the years, moving from EST-based resources [173,174,175,176] to novel NGS collections [106,108,110,177,178,179,180], which were implemented for collecting and disseminating expression data and associated tools. However, although the widespread interest in the field, none of these public resources is exclusively dedicated to plant responses to abiotic stress.

Many transcriptomics efforts also focused on the detection of non-coding RNAs, mainly based on high throughput sequencing technologies (see Ma et al. [181] as an example of review on this topic), or even on more advanced approaches for their in situ localization (see Lu et al. [182], Meng et al. [183] as non-exhaustive examples of opportunities in this field). This highlighted the active role played by non-coding RNAs circulating in plant tissues in physiological, pathological or in stress conditions [184,185,186] such as micro-RNAs [50], i.e., small non-coding RNAs of 19–23 nucleotide length, under abiotic stresses in many plant species. Trindade et al. [187] reported about the up-regulation in shoots and roots tissues of two conserved microRNAs, i.e., miR398 and miR404, in response to water deficit in *Medicago truncatula*. In two other studies, Gao et al. [188] detected a salinity and alkaline stress-related microRNA gene, i.e., osa-MIR393, whose over-expression can regulate rice salt and alkaline stress tolerance; and Zhang et al. [189] identified in tomato a drought stress related microRNA (Sly-miR169c), which, when overexpressed, produced transgenic plants with reduced stomatal opening, decreased transpiration rate, lowered leaf water loss and enhanced drought tolerance. Bokszczanin et al. [29] identified known and predicted novel miRNAs responsive or not to heat stress. Curaba et al. [190], finally, investigated the general role of miRNAs in targeting stress signaling pathways responsible for root development, leaf morphogenesis and stress response, reviewing about the role played by miRNAs in the crosstalk between phytohormone [191] signaling pathways. Small RNA collections are publicly available in RFAM [192] and miRbase [193] databases, while plant specific tools to support these specific efforts are described in the work of Srivastava et al. [194]. Examples of other plant dedicated platforms for related analyses are plantDARIO [195] and PsRobot [196]. Worthy to note, specific collections of smallRNAs are also available which are focused exclusively on plant responses to abiotic stresses (see paragraph “Dedicated web-based resources”).

## 4. Proteomics 

Enzymes and, more in general, proteins are fundamental for cell functionality and, consequently, plant responses to abiotic stresses may be triggered by protein contributions, since they play a key role in the establishment of phenotypic traits or in the plasticity of cell changes [197]. In this context, proteomics have become a powerful tool for the exploration of post-translational modifications of proteins, protein–protein interactions and, overall, for unravel molecular effects of perturbation caused in plant metabolism [32]. Proteomic studies, indeed, contribute to gain a deeper understanding of plant cell operability by identifying proteins affected in their accumulation, structure and, therefore, action, by abiotic stresses, or by related factors responsible for stress mitigation [198]. Thus, comparative proteomics analyses in physiological and stressed plants can help to identify protein effectors, targets and variability in interactive networks [199].

Historically, the first era of proteomics relied on the use of two-dimensional gel electrophoresis (2D-gel) [200], which is able to separate the proteins based on the isoelectric point (first dimension) and on the molecular mass (second dimension); subsequently, differentially accumulated proteins were subjected to mass spectrometry (MS) analysis [201]. More recently, the exploitation of the full potentialities offered by MS platforms originated the so called shotgun proteomics approach, with protein mixtures digested in smaller peptides, which are then separated and analyzed, respectively, by liquid chromatography (LC) and tandem mass spectrometry (MS/MS) for peptide identification [202] through data analysis tools [199]. Finally, sequenced peptides are assembled in order to gain, potentially, a whole proteome reconstruction using several bioinformatics tools such as IDPicker [203] and PAnalyzer [204].

Proteomic studies for stress responses have been thoroughly conducted in several plants, including *Arabidopsis thaliana*, *Triticum aestivum* (wheat), *Zea mays* (maize), *Oryza sativa* (rice), *Glycine max* (soybean), *Brassica napus* (oilseed rape), *Solanum tuberosum* (potato), *Solanum lycopersicum* (tomato) [205,206,207,208] and others (as also referred in [209]). These works highlighted the dynamic alterations in proteins and their different isoforms involved in signaling and regulatory pathways, transcription factors and protein–protein interactions; they clearly showed the importance of several factors including the genotype, the type and extent of the environmental stress, the protein subcellular localization, protein post-translational modifications (PTMs), etc.; and, finally, they allowed the identification of a series of proteins and enzymes putatively involved in the production of stress-related compounds and processes [206].

The proteomics study of Chen et al. [210] revealed differences in cold acclimation mechanisms in freezing-tolerant and freezing-sensitive cultivars of *Medicago sativa*. In particular, the results revealed that many proteins involved in photosynthesis, protein metabolism, energy metabolism, stress and redox were recruited for adaptation to cold stress. Ghabooli et al. [211] discussed molecular mechanisms underlying water stress tolerance induced by a fungus in barley. Their proteomics analysis resulted in the identification of 45 differentially accumulated proteins associated with photosynthesis, signal transduction and plant defense responses. Balbuena et al. [212] investigated how sunflower adjusts its metabolism during cold treatment through a comparative proteomic approach, which characterized 14 different patterns of expression across different sunflower lines, highlighting differential proteome responses to cold acclimation. The detected cold-responsive proteins were mostly involved in protein synthesis, energy (glycolysis) and defense processes. Other proteome analyses also hypothesized mechanisms for chilling attenuation in plants and cross-tolerance processes. More recently, Parrine et al. described proteome alterations on *Solanum lycopersicum* under high light stress and found a significant change in PSII complex proteins [213]; while a shotgun proteomic approach was applied by Jozefovicz et al. [214] to understand plant responses to nitrogen deficiency in two different varieties of *Solanum tuberosum*: in this work, differences between the two varieties were found to be strongly associated with protein catabolism, defense mechanisms as well as protein and amino acid synthesis metabolism.

Although their contribution in revealing biological mechanisms, as reported from the cited examples, there is a general difficulty to collect massive proteome data and organize them in accessible resources. Compared to other approaches, such as genomics or transcriptomics, public resources for the storage and dissemination of proteomics data are still poor, due in particular to the variety of different data types and experimental procedures that make the design of homogeneous collections very hard [215,216]. Moreover, tools for data mining and interpretation in proteomics may be more complex due to the great variety of signals, such as the possible number of targets for protein modifications and changes in interactions or interactors [217], the variety of analytical approaches [218,219] and of bioinformatics pipelines [220,221], and the associated statistical analyses [222,223], particularly in relation to peptide assembly and subsequent proteome reconstruction. These aspects are crucial to achieve informative and reliable findings, and also taking into account that a great number of fragmentation spectra in a typical shot-gun proteomics experiment remain unidentified and that this large portion of unidentified spectra could originate from unexpected modifications or natural peptide variants [224]. Consequently, proteomics data sharing, starting from raw data to value added information and associated mining tools, would require large investment, infrastructure and coordination [216]. In this context, the PAPPSO proteomic facility [225] has greatly contributed to the technological development to fill several gaps in the proteomics analysis pipeline: at this aim, tools enabling LC–MS alignment, peak extraction and detection (MassChroQ; [226]), peptide filtering, identification and grouping (X!TandemPipeline; [227]) and proteome data analysis (for instance, to highlight different protein isoforms and post translational modifications (PTMs)) and storage (PROTICdb; [228]) have been generated. Furthermore, a highly promising recent initiative aimed to overcome these limits is represented by the *Arabidopsis* proteome draft, providing a comprehensive and quantitative atlas on protein accumulation, modification (e.g., phosphorylation), regulation, localization, etc. [229], as well on their thermal stability [230] thus representing a valuable resource for the community.

Actually, the major public databases developed for proteomics data are The ProteomicsDB [231], The PRoteomics IDEntifications (PRIDE) database [232], PeptideAtlas [233] and the Global Proteome Machine Database (GPMDB) [234]. Unfortunately, PeptideAtlas does not contain experiments on plant species. On the contrary, PRIDE contains, up to now, 308 proteomics experiments on plant species, 72 of which are related to response to environmental stress. Although GPMDB contains proteomics data from plant species too, the web interface does not allow querying for lists of data, therefore we cannot refer on the current number of experiments related to abiotic stress. Two additional reference resources, Peptidome [235] and Tranche [236], did not have continuity due to the lack of funding, highlighting the difficulties in promoting and maintaining data sharing in proteomics field [216]. The Plant Proteomics Database (PPDB; [237]), although limited to *Arabidopsis* and maize studies, contains a series of proteomics experiments and tools for comparative and subcellular proteomics, biochemical pathways, etc. Interestingly, it allows the search and retrieve of specific proteins involved in the specific abiotic stress type, e.g., ozone, cold and drought [237].

More specialized databases, mainly focused on specific protein classes, are publicly available. Among them, ARAMEMNON [238] enables the identification of a series of stress-related integral membrane proteins in both monocots (maize, banana, rice and brachypodium) and dicots (*Arabidopsis*, poplar, grape, tomato and muskmelon). Finally, resources covering two important aspects in proteome studies, related to subcellular localization and PTMs, have also been developed: within the formers, plant subcellular proteome databases have also been generated at plastid (Plprot, [239]; AT_CHLORO, [240]) and cell wall (WallProtDB, [241]) levels, although they do not allow one to perform ad hoc searches for abiotic stress-specific peptides; while PMTs alterations, which have been shown to play a fundamental role in the abiotic stress-derived responses, can be investigated by a series of ad hoc databases. The Plant Phosphorylation database (P^3^DB, [242]) enables the investigation of changes in stress-induced phosphorylated proteins in six plant species (*Arabidopsis*, rapeseed, soybean, barrelclover, rice and maize) [242]. NetPhos 3.1 and PhosPhat, firstly launched in 1999 and 2007, and then further implemented in 2004 and 2010, respectively [243,244,245] allows predicting the pS, pT and pY sites as well retrieving information about kinase substrates, and is connected with a series of related databases (Pfam and MapMan) to infer information about the localization of experimental and predicted phosphorylation sites to known domains.

Specific collections for proteomics data focused exclusively on plant responses to stress are all described in the section “Dedicated web-based resources”.

## 5. Metabolomics 

Besides the transcriptome and proteome responses, abiotic stresses dramatically affect plant metabolic profiles [26,246,247]. The metabolome of a biological system, in fact, directly correlates with gene expression and protein accumulation of functional pathways that, in turn, reflects the organism responses to a vast range of status changes, such as the ones caused by an abiotic stress [32,248]. Moreover, the metabolic changes of an organism are those aspects of a stress response that are generally the most interesting ones in terms of phenotypic effects of the response [249,250]. Indeed, plant cells react to an adverse condition by remodeling their metabolism, finely tuning the presence/absence of specific metabolites, which can potentially represent biochemical markers of a particular stress. Thus, metabolomics, meant as the approaches to detect in a single analysis (“one shot”) the highest number of primary and secondary metabolites, enable the detection of alterations within the metabolic pattern, and to infer on associated variability in stress-inducible genes and proteins [32,251,252]. Currently, metabolomics is widely used for generating novel insights into plant responses to abiotic stress [53,253]. Several advances in high throughput techniques, moreover, increased the number of metabolomics studies [254].

Overall, plant metabolome is composed by two fractions: non-volatile metabolome, which includes metabolites soluble in hydro alcoholic solutions (polar, semi-polar compounds; amino acids, sugars, organic acids, phenylpropanoids, alkaloids, etc.) or in organic solvents (non-polar; lipids, isoprenoids, etc.) [255,256]; volatile metabolome, comprising of semi-volatile (diterpenes) and volatile (alcohols, ethers, esters, mono- and sesqui-terpenes, etc.) molecules [257,258]. Basically, in metabolomics two approaches are used to achieve a global metabolic profiling: the “untargeted” and the “targeted” approaches [259,260,261]. In the former [262], all the mass chromatograms of an experiment are processed by tools allowing comparisons and retrieving all the differentially accumulated ions (irrespectively of their identity) in the samples under study, for instance stressed versus control, which are then processed using bioinformatics approaches, such as PCA or other multivariate analyses, in order to obtain a “metabolic finger-print”, which is characteristic of the biological process under investigation. The “targeted” approach, based on the use of public, custom or ad hoc metabolomics databases, enables the detection and quantification of known primary and secondary metabolites.

The first metabolomics studies of plant responses to cold stress were conducted in *Arabidopsis* [263,264], revealing that its metabolome is extensively reconfigured in response to low temperature, particularly at amino acid, organic acid and polyamine levels. Other later publications have been: the work of Urano et al. [265], in which a metabolomics characterization was carried out under drought on *Arabidopsis* leaves of wild type and a knockout mutant in the *NCED3* gene, which plays a key role in the dehydration-inducible biosynthesis of abscisic acid (ABA) in maize. Interestingly, this study allowed unraveling a functional role for amino acids and raffinose in the stress acclimation. Skirycz et al. [266] performed a metabolite profiling of *Arabidopsis* leaves under osmotic stress, showing that proliferation and expansion were regulated by common regulatory circuits, involving ethylene and gibberellins, but not ABA. Metabolomics studies on drought responses of leaf tissues were conducted also in tomato [267], trying to assess the contribution of loci with over-dominant effect to the yield and fitness; in wheat [268], identifying compounds (again, particularly amino acids and organic acids) that differed in three distinct cultivars characterized by different levels of tolerance to drought; and in maize [269], reporting on six different maize hybrids for their responses to drought stress, mainly associated to changes in amino acids, sugars, sugar alcohols and intermediates of the TCA cycle. In a different attempt, van Dongen et al. [270] described the metabolic responses under anoxic conditions due to flooding stress in *Arabidopsis* roots. In particular, through a metabolite profiling approach, the authors inferred that genes that were down-regulated mainly encoded proteins involved in metabolic energy-consuming processes. Tohge et al. [271], finally, analyzed the transcriptional and metabolic programs under light stress, characterizing the genes in the UV-B signaling cascade, and reviewing also the pathways mainly responding to UV-B exposure.

Due to the importance of salinity stress in agriculture, there are a lot of metabolomics studies on crop species, such as *Arabidopsis* [272,273,274], rice [275], tomato [276,277], grapevine [278], sea lavender [279], poplar [280] and the legume genus *Lotus* [281,282], revealing the compounds involved in resistance and tolerance to salt stress, thus assisting the identification of candidate genes associated to abiotic stress responses. More in detail, these studies evidenced the involvement of a series of sugars, chorismic acid derivatives, phenylpropanoids (phenolic acids as ferulic and vanillic acids) and down-stream metabolic products. Additionally, investigations on effects of nutrient limitation on plants often exploited metabolomics approaches, such as *Arabidopsis* [283,284,285,286,287,288], barley [289], tomato [290] and the common bean [291,292], analyzing the effects of the lack, among others, of carbon, nitrogen, sulfur, phosphate and carbohydrates, with the subsequent remetabolization of proteins and lipids as alternative substrates in respiration and other cellular processes. *Arabidopsis* was the object also of many metabolomics studies on responses to oxidative stress [293,294,295,296,297], in which specific inhibitions of enzymes of the tricarboxylic acid (TCA) cycle (aconitase and isocitrate dehydrogenase) and inhibition of glycolysis and TCA cycle flux emerged. Ishikawa et al. [298], moreover, analyzed the entire rice metabolome in response to oxidative stress; their results indicated that tolerance to oxidative stress, obtained by attenuation of cell death and growth inhibition processes, is due to an enhanced capacity of metabolic acclimation. Frequently, the comparison between susceptible and tolerant genetic materials is a useful tool to disclose the molecular/biochemical changes occurring as result of the abiotic stress: for instance, the magnesium nutritional deficiency in grapevine rootstock, drastically affecting growth and plant productivity, has been associated to dramatic changes at transcript and metabolite level, mainly altering cell wall components, antioxidants and secondary metabolites as alkaloids, terpenoids and phenylpropanoids [299].

More recently, the effects of drought and high temperatures, alone or in combination, have been investigated in a group of susceptible/tolerant potato cultivars [300], which allowed identifying a set of mechanisms negatively affecting photosynthesis (with a simultaneous increase of the non-photochemical quenching), and its compensation by a consequent reorganization of cell metabolism.

Despite the wide variety of approaches, some of which are here briefly depicted, it is not the purpose of this review to present in detail different metabolomics efforts for plant abiotic stress, which range from gas chromatography–mass spectrometry (GC–MS) and liquid chromatography coupled to high resolution mass spectrometry (LC)–HRMS to capillary electrophoresis (CE)–MS and nuclear magnetic resonance (NMR) spectroscopy [301]. Our main aim, instead, is to examine related bioinformatics data resources in the field. Interestingly, only one web-based platform collecting metabolome data related also to abiotic stress responses in plants is currently available: MetaboLights, a database for metabolomics experiments and derived information [302]. Querying this resource with the “abiotic stress” keyword determined 54 experiments on a wide variety of species ranging from prokaryotes to mammals, 30 of which are on plants. In this frame, the other two metabolomics resources fully dedicated to plants are the Plant Metabolome Database (PMD) [303] and PlantMetabolomics.org [304]. These two resources, however, appear out of service according to our attempt to access the data. Although not specific for abiotic stress and the plant field, valuable and routinely updated resources are the Human Metabolome Database (HMD, [305]), composed by four sections, DrugBank, T3DB, SMPDB and FooDV, and including both polar and non-polar compounds, organized in metabocards, which are hyperlinked to other databases (KEGG, PubChem, MetaCyc, ChEBI, PDB, UniProt and GenBank); Metlin, computer and mobile-based platform developed in 2003, and now including either small molecules, drugs and peptides, and provides experimental and in silico fragmentation as well as an ID search based on an accurate precursor and fragment masses, and on MS/MS spectrum match; similarly to HMD, it also includes links and information for any of their 960,000 compounds, with systematic name, structure, elemental formula, mass, CAS number, other database link (KEGG, HMDB, PubChem, etc.), commercial availability, etc. [306]; Massbank [307], database of mass spectra of known and unknown compounds, which has been is the first public repository of mass spectral data, enabling numerous search options based on compound name and/or exact mass and/or formula, and information about the MS platform type, as well the MS filter type (MS, MS/MS, MS3, etc.) [307].

An interesting tool able to predict the relationships between gene expression and metabolite levels, based on their contemporary responses to abiotic stresses, is represented by a command line software [308]. The objective of this software is to predict pathways containing gene and metabolites, which interact due to their coresponses to stress. In the same context, although not specifically related to abiotic stress responses, is the Knapsack family database [309], in which metabolite data (search according to the accurate mass, molecular formula, metabolite name or MS ionization mode) are tightly interconnected to information about the species and the geographical area in which they have been found, and their biological activities [310], an aspect of interest for the study of many biological processes, including abiotic stress responses; as further implementation, the 3D structure for each compound have been included in the Knapsack DB using the Merck Molecular Force Field (MMFF94), providing novel opportunity for the identification of new and potentially unexpected binding sites for target proteins by docking studies, or for the estimation of biological activities using 3D-QSAR [311].

Overall, metabolomics collections are characterized by a higher extent of constraints due mostly to the limitations in terms of data uniformity, mainly attributed to the use of different technologies and experimental conditions. In addition, metabolomics trust is negatively affected by the great heterogeneity of plant molecules, particularly the secondary metabolites, which reduces the possibility to unambiguously identify a target compound. In this context, relevant bottlenecks are represented by the lack of orthology-based analyses, i.e., analyses based on sharable reference information, allowing, as an example, the possibility to use an accurate molecular mass of a secondary metabolite to look for and to confirm the presence of the same metabolite in other plant systems, which is a typical comparative approach largely exploited in all the other omics [312,313].

Thus, metabolite annotation represents a key step towards the ability to decipher the set of compounds mostly associated to a specific process (e.g., an abiotic stress). This aspect is partially addressed in the “targeted” approach, where metabolite identification is achieved by a series of approaches including standard confirmation (if available, and representing the only unequivocal approach to validate the identity of a molecule) and comparison between theoretical and experimental MS/MS fragmentation; even more intricate is the “untargeted” metabolomics field, which requires the availability of commercial (provided by any mass spectrometry company) or freeware tools, able to extract the accumulated metabolites in a MS chromatogram, which are subsequently subjected to an annotation pipeline to be identified. Within the latter, MZmine 2 is a tool comprised by a great number of modules allowing MS data analysis from raw data processing and peak detection and identification until visualization and bioinformatics/statistical analyses [314]; MetAlign and MSClust, developed at WUR, are two freely available software performing GC– and LC–MS preprocessing by isolating the detected ions in the MS run through a series of operations (accurate mass calculations, baseline corrections, peak-picking, saturation and mass-peak artifact filtering); subsequently, they are processed by an unsupervised clustering approach followed by extraction of putative metabolite mass spectra [315]. More recently, MetFrag [316] is a powerful web-tool able to interrogate different databases (as Chemspider, PubChem, KEGG, etc.), and to perform a sequential annotation pipeline based on MS/MS fragmentation pattern.

An exception with respect to the identification biases of LC–MS platforms is represented by nuclear magnetic resonance (NMR) technology, which allows unequivocal metabolite identification, although its typical lower sensitivity does not allow measuring a large number of compounds, compared to LC–MS and GC–MS [317,318]. Moreover, the absence of an adequate biological knowledge on the measured metabolites (for instance, the physiological processes in which they are accumulated) makes the generation of functional resources that could specifically indicate the set of metabolites accumulated hard. To date, in fact, all the metabolomics databases we could overview, display a satisfying level of details in terms of chemical characteristics of the metabolites; furthermore, for some of them, the specific association to the pathway in which they are synthesized is reported. Anyhow, and unfortunately, they do not provide any information about the biological processes in which the target metabolites are accumulated, for instance in response to a specific or more general stresses.

## 6. Data Integration and Mining 

The generation of massive amounts of data from different -omics approaches results in an ever-growing need of powerful and informative bioinformatics tools, able to provide easily accessible and efficient integrative views to the enriched information that data can deliver. From this viewpoint, it is remarkable to cite the major platforms or resources for investigating processes and components in terms of genes, proteins and metabolites acting in specific processes and functional pathways.

Among reference resources providing metabolic and biological process information, we consider three widely used databases, i.e., KEGG [319], Reactome [320] and MetaCyc [321]. Querying for “abiotic stress” in the main page of the KEGG database resulted in only five *Oryza sativa* (rice) proteins involved in responses to abiotic stress. However, querying for “abiotic stress” exclusively in the pathways section of KEGG resulted in only one MAPK signaling pathway in plants (available at [322], which includes, among others, responses to cold, salt, drought and osmotic stress. Querying for “abiotic stress” in the main page of the Reactome database retrieved seven hits in both *Arabidopsis* and rice species, including, among others, pathways related to cellular response to heat and oxidative stress (available at [323]). A similar search in MetaCyc retrieved no results. A deeper manual search within MetaCyc enabled to detect only single reactions involved in responses, among others, to heat, cold, oxidative and starvation stresses (results available at [324]).

A fundamental resource for gene/protein studies and comprehension of their role in processes and networks is represented by the gene ontology (GO) terms. One of the main uses of the GO is to perform an enrichment analysis (GOEA) on a group of genes, transcripts and proteins. For example, given a set of genes that are up- or down- regulated under certain conditions, an enrichment analysis will find which GO terms are over or under-represented with respect to a reference list (for instance the whole transcriptome), using annotations for that gene set. AmiGO [325] is probably the most used platform for searching and browsing the Gene Ontology database. A query like “abiotic stress” in AmiGO resulted in five gene ontology terms, synonyms or definitions and 12,800 genes or gene products associated with GO terms (results available at [326]). In particular, among the five detected GO terms, two GOs are now obsolete (“abiotic stress sensitivity” and “abiotic stress sensitivity value”) while three GOs, i.e., “response to stress”, the “cellular response to abiotic stimulus” and “response to abiotic stimulus”, are each the subset of the other. These hierarchical annotations often may be non-specific and cause redundancy that can lead to a misinterpretation of the enrichment results. Thus, all the current limitations, which are also emerging by our overview, highlight that, despite the relevance of these resources in molecular biology, focused and coordinated efforts are still required to integrate and make more curated information available to the plant scientific community, to support the appropriate mining of the effective information these systems may provide. 

Another important resource for GO enrichment analysis studies is blast2GO [327], which allows self-independent annotations of datasets by comparisons with data from InterPro, enzyme codes, KEGG pathways, GO direct acyclic graphs (DAGs) and GOSlim. Blast2Go is a powerful tool in particular to obtain information about neo-sequenced transcriptomes that still do not have a curated annotation and need to be processed to support investigations on the biological information content they represent. More recently, an integrated web-based GO analysis toolkit for the agricultural community named AgriGO [328] was developed. AgriGO can perform GOEA on 45 species and 292 datatypes; additional bioinformatics analyses include SEA (singular enrichment analysis), PAGE (parametric analysis of gene set enrichment), BLAST4ID (transfer IDs by BLAST) and SEACOMPARE (cross comparison of SEA) [328]. Subsequently, a new updated release of this tool, called AgriGO2.0 [329], has been made publicly available. AgriGO2 is characterized by a series of implementation, including the number of supporting species (394) and datatypes (865). Furthermore, an improved computational efficiency, comprising the batch analysis and *p*-value distribution (PVD), as well tools like direct acyclic graph (DAG) and scatter plots and a general higher user-friendliness of the web pages were achieved [329]. 

A great boost in the promotion of a better comprehension of multilevel variations of the biological systems under investigation has been provided by the MapMan software [330], which allows one to “map” transcripts, proteins and metabolites on cellular pathways and processes, which can be directly downloaded by the software website, as well as being self-created by the users. One of the main advantages of MapMan is its flexible and open nature, with the possibility, for any scientist, to generate specific maps according to research interests, simply by uploading pathway images and input data, as well as by modifying the usual input file containing all the information to plot genes/proteins/metabolites. Historically, MapMan was originally developed for transcriptomics studies but, more recently and due to its versatile nature, it was converted into a tool able to investigate proteins and metabolites fluctuations [331]. With the aim to improve data elucidation, MapMan was strongly improved at the gene functional annotation level by the generation of GOMapMan [332], which provides a series of functionalities as gene annotations for plant species through the integration of ortholog group information, increased knowledge about gene functions via literature interrogation, etc. In this context, the simultaneous and combined use of this kind of tools can improve the characterization of transcripts and proteins, which can be associated to abiotic stress responses. In relation to the topic of this review, it is worth underlining that, to date, a series of preassembled maps are available for some plant species in the MapMan website (available at [333]) covering biotic/abiotic stresses (named “Biotic Stress”, “R_stress” and “R_nutrients”), although, a better extent of detail would be still needed. 

Another powerful tool to elucidate and unravel molecular–biochemical mechanisms underlying plant biological processes and, thus, abiotic stress responses too, is represented by the use of network theory approaches [334] and mining tools exploiting mathematical indexes, such as correlation coefficients (Pearson, Spearman, etc.). This tool permits to identify candidate elements (transcripts, proteins and metabolites) that are coexpressed/accumulated in specific functional events. In this context, a useful and appealing way to visualize data is provided by the so-called correlation matrices and by network graphs, which can be generated using several widespread tools. Cytoscape is the most known software to investigate correlation networks [335]. It is an open source project, which can support the understanding of complex -omics dataset by representing each element (transcript, protein, metabolite, etc.) as a node, with edges connecting the nodes and representing correlative relationships between them. Cytoscape is supported by an ever-growing community, working on software implementation and improvement [336], and contributing several additional plugins [337] to investigate different aspects as statistics, network topology, pathway annotation, etc. A coupled transcriptomics/cytoscape-based approach, as an example, has been exploited, to study the origin and the evolution of stress responses (more in the detail, to abscisic acid (ABA), cold, drought and salt treatments) in *Physcomitrella patens* [338]. Overall, 9668 differential expressed genes in response to stresses were identified in this work, and further comparison between *P. patens* and unicellular algae, vascular and flowering plants evidenced a series of genetic changes associated with the evolutionary movement to land. In a more recent work [339], proteomic data on soybean leaves subjected to drought and heat stresses were analyzed using correlation networks, highlighting a series of protein interactions involved in RuBisCO activity, electron transport and carbon fixation, as well as a group of EF-Tu proteins, which could be directly related to heat stress tolerance mechanisms.

## 7. Dedicated Web Based Resources 

Public web-based accessible databases and platforms dedicated to stress response in plants are also available. They are essential to organize results and all the necessary information from different levels of investigations, to make it available to all the interested scientific community. 

In this context, Plantstress [340] is a general web-based resource of information, a consultation facility and a source for professional update on the most important issues on plant environmental abiotic stress.

The Plant Stress Gene Database [341] is a database of 259 genes from 11 plant species involved in stress conditions. Through the web page, it is possible to search information querying by species, gene ID or function. Moreover, it is also possible to obtain information about paralog or ortholog genes among the species included in the database.

For the reference model in plant biology, The *Arabidopsis thaliana* Stress Responsive Gene Database (ASRGD) is available [342]. It represents a public collection of genes related to stress responses, based exclusively on manual curated stress tolerance genes associated with *A. thaliana*. This resource includes 637 genes related to about 50 different stress conditions, exploitable by keywords or by stress type (i.e., osmotic, heat, etc.).

Similarly, at the protein level, The Plant Stress Protein Database (PSPDB) [343] is a public resource that covers 2064 manually curated plant stress proteins from 134 plant species, highlighting their functional roles under the pressure of 30 different types of biotic and abiotic stresses. It is possible to retrieve information from the database searching by gene, species, keyword, citation, gene families and taxonomic classification. Another resource in the field of proteomics for plant responses to general stresses (biotic and abiotic) is PlantPReS [344], which comprises of more than 20,413 entries from 456 manually curated articles, and more than 10,600 unique stress responsive proteins. PlantPReS represents a very valuable resource for the plant stress community, due to a user-friendly interface and several analysis tools, as search engine, gene ontology, cross-referencing and expression patterns of target proteins involved in a stress response.

Furthermore, ad hoc resources for specific stress types are also available. For instance, the DroughtDB [345] is a public resource that includes manually curated genes involved in the drought stress response, providing detailed information about computed ortholog genes in nine model and crop plants.

On the other side, specialized databases have also been generated on the basis of the molecular and functional roles. For example, The Stress Responsive Transcription Factor Database (STIFDB v.2) [346], useful for targeted as well as high-throughput experimental and computational studies to investigate stress responses in *A. thaliana* and in *O. sativa*, currently, has more than 38 thousands associations of stress signals, stress-responsive genes and transcription factor binding sites, predicted using the stress-responsive transcription factor (STIF) algorithm based on an HMM model. The user can interrogate the resource by gene name, chromosome, transcription factor and/or stress signal. Although limited to users working in the rice–water stress community, the RiceSRTFDB [347] is a database of rice transcription factors containing expression patterns, cis-regulatory element and mutant information to facilitate gene function analysis during drought and salinity stress conditions obtained from microarray experiments. The website page offers an Expression Viewer that allows one to check the differential expression of the selected genes in salinity or drought stresses.

PASmiR [348] is a database collecting information on miRNA molecular regulation in plant abiotic stress, including data from about 200 published studies, which represent 1038 regulatory relationships between 682 miRNAs and 35 different type of abiotic stresses in 33 plant species. The query system allows querying by miRNA name, species or/and type of abiotic stress and the user has the possibility to download all the data included in the database.

To date, no metabolomics databases, specifically focused on abiotic stresses are available, although, at least at the repository level, a series of data collected under different stress conditions in *Arabidopsis*, *Brachypodium distachyon* and wheat are accessible at PRIME, the Platform for RIKEN Metabolomics, which is a Web-based service for metabolomics and transcriptomics analyses [349,350]. An interesting attempt to better integrate different metabolomics resources and studies, as well to connect metabolic phenotypes to different genetic materials unraveling biological processes at both biochemical and molecular levels, is represented by PhenoMeter (PM), which can use metabolite response patterns as queries and searches the MetaPhen database for responses that are statistically significantly similar or inverse, thus highlighting functional links [351]. The effectiveness of this approach was also confirmed by using specific case studies and cross-matching different data based on the investigation of responses of *Lotus japonicus* to salt stress [281] and of *A. thaliana* to sulfur deficiency [352] and cold and heat stress [264].

The list of the resources here presented (Table 6), in comparison to the variety of studies on different plants and stress types, and the limited number of integrated platforms dedicated to abiotic stresses, highlights that, although the spreading of advanced molecular technologies and novel challenging projects in the field (e.g., [353]), coordinated and completed efforts that could provide data and information resources for further investigations and mining in the field are still strongly required [354].

## 8. Conclusions

Research on abiotic stress responses covers a relevant field of interest in plant sciences since this specific -omics knowledge is consequently essential to develop improved crop plants in terms of quality and productivity, showing enhanced level of abiotic stress tolerance and possibly disease resistance.

The bioinformatics in the post-genomics era is revolutionizing the way the molecular experiments can be designed, thus favoring comprehensive views on different levels of biological functionalities, and providing substantial contributions to increase the scientific knowledge while adding new perspectives to programs for enhancing stress tolerance in crops.

The actual spreading of -omics technologies is driving the expansion of the available collections also on abiotic stress in plants, although the intrinsic specificity and heterogeneity of such analyses. However, we are still in an early stage. Our overview on the many different efforts that have been undertaken worldwide was also aimed to highlight that, although the spreading of the activities in the field, data from -omics technologies and bioinformatics efforts, as well as coordination in data providing and maintenance are still highly required to organize useful data collections and to offer consistent and comparable datasets, useful to suitable computational benchworks for appropriate investigations. However, the numerous opportunities to answer to different scientific aims, focused on different samples (e.g., species or genotypes), based on different experimental specificities, offer amounts of resulting collections and resources, which are challenging the possibility to support useful integration and appropriate comparative efforts, permitting appropriate, multilevel analyses, proper data organization, annotation and integration in web accessible resources, as well as data curation and user-friendly accessibility are indeed still highly demanded, opening the field to the new challenge of integrative biology. There is need to cover coherent multilevel investigations, from genomics to metabolomics, also considering epigenomics, in the same systems and under similar stimuli, favoring extensive comparative analyses. As example comparisons among data in physiological conditions, or in different tissues or genotypes, need appropriate platforms for flexible investigations, permitting holistic views or insights into specific molecular processes even to non-expert users. The investigation of heterogeneous stimuli on the same systems, if comparable, can also favor the detection of similarities and peculiarities of the different responses and further highlight the complexity of plant molecular responses.

The general usefulness of bioinformatics web based resources to manage and decipher the information hidden in the large amount of biological collections provided by current -omics methodologies is strongly linked to data representativeness, consistency, quality and updating. This affects the value added information that can be delivered, the opportunities of integration with other resources and the user-friendly accessibility. Beyond a series of aspects including the variety of scientific fields, the heterogeneous performances of different experimental approaches, the technical and methodological specificities and the limits of the bioinformatics data processing overviewed in this review, the availability of large amount of varied multifaceted data also requires the big challenge of distribution, maintenance and coherent versioning [355].

Although many efforts are arising, the common framework that will support challenging scientific applications is therefore coordination, accessibility and integration.

## Figures and Tables

**Table 1:** Gene annotation version of model plants or plants of agronomic interest as reported in public web accessible platforms.

Species	Ensembl Plants [101]	NCBI [97]	Phytozome [103]	PlantGDB [102]	Plaza [104]
*Amborella*	AMTR1.0	GCF_000471905.2 (AMTR1.0)	*Amborella trichopoda* v1.0	NA	JGI v1.0 [113]
*Arabidopsis*	TAIR 10 [107]	TAIR 10 [107]	TAIR 10 [107]	TAIR 10 [107]	Araport11
Bread wheat	IWGSC	GCA_002220415.3 (Triticum_4)	*Triticum aestivum* v2.2	NA	IWGSC1.1
Banana	*Musa acuminata* DH-Pahang v1 (ASM31385v1)	GCF_000313855.2 (ASM31385v2)	*Musa acuminata* DH-Pahang v1	NA	*Musa acuminata* DH-Pahang v2
Clementine	*Citrus_clementina*_v1.0	GCA_000493195.1 (*Citrus_clementina*_v1.0)	Citrus_clementina_v1.0	NA	Citrus_clementina_v1.0
Cocoa	Criollo_cocoa_genome V2.44	GCF_000208745.1 (Criollo_cocoa_genome_V2)	C. Matina v1.1	NA	GCF_000403535.1
Grapevine	V1 Cribi [114]	GCF_000003745.3 (12x)	V2 Genoscope [115]	V2 Genoscope [115]	V2 Genoscope [115]
Jojoba	NA	GCA_900322235.1 (ASM90032223v1)	NA	NA	NA
Maize	B73_RefGen_v4	GCF_000005005.2 (B73_RefGen_v4)	B73_RefGen_v3	B73_RefGen_v2	B73_RefGen_v4
Oilseed rape	AST_PRJEB5043_v1	GCA_000686985.2 (Bra_napus_v2.0)	NA	NA	NA
Pepper	NA	GCF_000710875.1 (Pepper Zunla 1 Ref_v1.0)	NA	NA	Pepper Genome v.2.0
Potato	SolTub_3.0	GCF_000226075.1 (SolTub_3.0)	PGSC v. 4.03 [109]	PGSC v.3 2.1.10 [109]	PGSC v. 4.03 [109]
Rice	RGAP 7	GCF_001433935.1 (RGAP 7)	RGAP 7	RGAP 7	RGAP 7
Sorghum	Sbi3.1.1	GCF_000003195.3 (*Sorghum_bicolor*_NCBIv3)	Sbi3.1.1	Sbi1.4	Sbi3.1.1
Soybean	Wm82.a2.v1	GCF_000004515.5 (*Glycine_max*_v2.1)	Wm82.a2.v1	Wm82.a2.v1	Wm82.a2.v1
Sweet orange	NA	GCF_000317415.1 (Csi_valencia_1.0)	JGI v1 [113]	NA	NA
*Thellungiella halophila* (*Eutrema salsugineum*)	NA	GCA_000478725.1 (Eutsalg1_0)	*Eutrema salsugineum* v1.0	NA	NA
*Thellungiella parvula (Eutrema parvulum*)	NA	GCA_000218505.1 (*Eutrema_parvulum*_v01)	NA	NA	TpV84
Tomato	iTAG v.3.0 [108]	GCF_000188115.4 (SL3.0)	iTAG v. 2.4 [108]	NA	iTAG v. 2.4 [108]

**Table 2:** Summary of genes including “abiotic stress” and “drought stress” in their functional annotation in NCBI Gene and in RefSeq databases.

	“Abiotic Stress”		“Drought Stress”	
Species	NCBI Gene Counts	NCBI RefSeq Counts	NCBI Gene Counts	NCBI RefSeq Counts
*Arabidopsis thaliana*	132	63	102	52
*Beta vulgaris*	1	-	-	-
*Brachypodium distachyon*	-	-	1	186
*Brassica napus*	-	-	1	2
*Capsicum annuum*	-	5	2	3
*Chlamydomonas reinhardtii*	1	2	-	-
*Cicer arietinum*	-	1	-	28
*Cucumis melo*	1	-	-	-
*Cucumis sativus*	1	2	-	-
*Elaeis guineensis*	1	-	-	-
*Eutrema salsugineum*	1	-	-	-
*Glycine max*	3	76	7	34
*Gossypium hirsutum*	1	2	-	1
*Hordeum vulgare*	1	-	-	-
*Jatropha curcas*	-	15	-	1
*Malus domestica*	2	1	-	-
*Manihot esculenta*	-	4	-	-
*Musa acuminata*	1	-	-	-
*Nicotiana tabacum*	1	3	-	-
*Oryza sativa*	9	-	2	-
*Populus euphratica*	-	1	-	-
*Prunus avium*	-	-	2	-
*Prunus persica*	1	1	-	-
*Ricinus communis*	-	-	1	1
*Solanum lycopersicum*	11	16	6	13
*Solanum tuberosum*	1	6	-	-
*Triticum aestivum*	6	-	-	-
*Vigna radiata*	1	-	-	-
*Vitis vinifera*	1	-	1	6
*Zea mays*	5	33	6	108
**Total**	**182**	**231**	**141**	**435**

**Table 3:** Summary of all the “abiotic stress” microarray experiments available in Gene Expression Omnibus (GEO) and Array Express databases.

Species	GEO	ArrayExpress
*Arabidopsis thaliana*	140	78
*Brachypodium distachyon*	-	1
*Brassica juncea*	-	1
*Carica papaya*	-	1
*Capsicum annum*	4	-
*Cicer arietinum*	4	1
*Ectocarpus siliculosus*	-	1
*Euphorbia esula*	-	1
*Glycine max*	10	4
*Gossypium hirsutum*	4	3
*Helianthus annuus*	7	4
*Hordeum vulgare*	69	3
*Ipomoea batatas*	-	1
*Lotus* sp.	-	1
*Malus domestica*	-	1
*Medicago truncatula*	5	1
*Nicotiana tabacum*	5	1
*Orchesella cincta*	-	1
*Oryza sativa*	103	26
*Panax ginseng*	-	1
*Petunia × hybrida*	6	1
*Poncirus trifoliata*	-	1
*Populus sp*.	10	2
*Populus tremula × Populus alba*	4	1
*Populus × canadensis*	6	-
*Pyrus pyrifolia*	-	1
*Solanum lycopersicum*	13	4
*Solanum melongena*	4	-
*Solanum tuberosum*	18	2
*Sorghum bicolor*	-	1
*Thellungiella*	-	1
*Triticum aestivum*	9	1
*Vigna unguiculata*	-	1
*Vitis vinifera*	8	3
*Zea mays*	15	7
**Total**	**444**	**157**

**Table 4:** “Abiotic stress”-related expressed sequence tag (EST) libraries per each plant species available in dbEST.

Species	N. of ESTs	N. of EST Libraries
*Agave sisalana*	14	1
*Arachis hypogaea*	30	2
*Brassica napus*	5856	5
*Catharanthus roseus*	4	1
*Cicer arietinum*	1	1
*Coffea arabica*	41,985	28
*Cucumis sativus*	7	1
*Fragaria vesca*	41,430	5
*Gossypium arboreum*	778	1
*Haberlea rhodopensis*	34	1
*Landoltia punctata*	7	2
*Opuntia streptacantha*	329	1
*Oryza sativa Indica Group*	88	3
*Oryza sativa Japonica Group*	177	1
*Persicaria minor*	4	1
*Pisum nigrum*	1	1
*Pisum sativum*	10	2
*Selaginella lepidophylla*	1046	1
*Solanum tuberosum*	20,758	1
*Triticum aestivum*	81,086	13
*Vitis vinifera*	16,492	2
*Withania somnifera*	1	1
**Total**	**210,138**	**75**

**Table 5:** “Abiotic stress”-related accessions per plant species/instrument available in sequence read archive (SRA) database.

Organism Name	Instrument	Library Strategy	Counts
*Arabidopsis thaliana*	Illumina HiSeq 2000	ncRNA-Seq	39
*Arabidopsis thaliana*	Illumina HiSeq 2000	RNA-Seq	4
*Arabidopsis thaliana*	Illumina HiSeq 2500	RNA-Seq	14
*Arabidopsis thaliana*	NextSeq 500	RNA-Seq	33
*Avicennia marina*	NextSeq 500	miRNA-Seq	3
*Boechera gunnisoniana*	Illumina HiSeq 2000	RNA-Seq	1
*Boechera stricta*	Illumina HiSeq 2000	RNA-Seq	1
*Brassica juncea*	Illumina Genome Analyzer IIx	RNA-Seq	6
*Brassica napus*	Illumina HiSeq 2000	RNA-Seq	12
*Camellia sinensis var. sinensis*	Illumina Genome Analyzer II	miRNA-Seq	1
*Capsicum annuum*	Illumina HiSeq 2500	RNA-Seq	78
*Cicer arietinum*	Illumina Genome Analyzer IIx	RNA-Seq	8
*Coffea canephora*	AB 3730xL Genetic Analyzer	CLONE	1
*Cymodocea nodosa*	Illumina HiSeq 2500	RNA-Seq	12
*Eleusine coracana*	Illumina HiSeq 2000	RNA-Seq	4
*Glycine max*	Illumina HiSeq 2000	RNA-Seq	4
*Helianthus annuus*	HiSeq X Ten	RNA-Seq	1
*Helianthus annuus*	Illumina HiSeq 4000	RNA-Seq	96
*Hordeum vulgare* subsp. *vulgare*	Illumina HiSeq 4000	RNA-Seq	32
*Hydrilla verticillata*	454 GS FLX Titanium	RNA-Seq	2
*Ipomoea trifida*	Illumina HiSeq 2500	RNA-Seq	15
*Ipomoea triloba*	Illumina HiSeq 2500	RNA-Seq	15
*Medicago ruthenica*	Illumina Genome Analyzer II	RNA-Seq	1
*Medicago sativa*	Illumina HiSeq 2000	RNA-Seq	1
*Medicago truncatula*	Illumina Genome Analyzer II	RNA-Seq	6
*Mesembryanthemum crystallinum*	454 GS FLX Titanium	RNA-Seq	2
*Oryza sativa* Japonica Group	Illumina Genome Analyzer IIx	RNA-Seq	18
*Oryza sativa* Japonica Group	Illumina Genome Analyzer	OTHER	9
*Oryza sativa* Japonica Group	Illumina HiSeq 4000	RNA-Seq	66
*Piper nigrum*	Illumina HiSeq 2000	RNA-Seq	1
*Prunus armeniaca*	Illumina HiSeq 2500	RNA-Seq	60
*Prunus armeniaca*	NextSeq 500	RNA-Seq	60
*Prunus persica*	Illumina HiSeq 2500	RNA-Seq	138
*Quercus suber*	454 GS FLX Titanium	OTHER	4
*Solanum lycopersicum*	Illumina HiSeq 2000	ncRNA-Seq	2
*Sorghum bicolor*	Illumina HiSeq 2500	RNA-Seq	24
*Triticum aestivum*	454 GS FLX Titanium	RNA-Seq	2
*Triticum aestivum*	Illumina HiSeq 2000	RNA-Seq	4
*Triticum aestivum*	Illumina HiSeq 2500	RNA-Seq	4
*Zea mays*	Illumina HiSeq 2000	RNA-Seq	32
**Total**			**816**

**Table 6:** “Abiotic stress”-related databases and platforms publicly available.

Plant Stress Dedicated Resources	Year
Arabidopsis thaliana Stress Responsive Gene Database (ASRGD) [342]	2013
DroughtDB [345]	2015
PASmiR [348]	2013
PlantPReS [344]	2016
Plantstress.com [340]	2007–2017
Plant Stress Gene Database [341]	2011
Plant Stress Protein Database (PSPDB) [343]	2014
RiceSRTFDB [347]	2013
Stress Responsive Transcription Factor Database (STIFDB v.2) [346]	2013
